# A Game Theoretic Framework for Analyzing Re-Identification Risk

**DOI:** 10.1371/journal.pone.0120592

**Published:** 2015-03-25

**Authors:** Zhiyu Wan, Yevgeniy Vorobeychik, Weiyi Xia, Ellen Wright Clayton, Murat Kantarcioglu, Ranjit Ganta, Raymond Heatherly, Bradley A. Malin

**Affiliations:** 1 Department of Electrical Engineering and Computer Science, Vanderbilt University, Nashville, Tennessee, United States of America; 2 Center for Biomedical Ethics and Society, Vanderbilt University, Tennessee, United States of America; 3 Department of Computer Science, University of Texas at Dallas, Richardson, Texas, United States of America; 4 Department of Biomedical Informatics, Vanderbilt University, Nashville, Tennessee, United States of America; Tianjin University of Technology, CHINA

## Abstract

Given the potential wealth of insights in personal data the big databases can provide, many organizations aim to share data while protecting privacy by sharing de-identified data, but are concerned because various demonstrations show such data can be re-identified. Yet these investigations focus on how attacks can be perpetrated, not the likelihood they will be realized. This paper introduces a game theoretic framework that enables a publisher to balance re-identification risk with the value of sharing data, leveraging a natural assumption that a recipient only attempts re-identification if its potential gains outweigh the costs. We apply the framework to a real case study, where the value of the data to the publisher is the actual grant funding dollar amounts from a national sponsor and the re-identification gain of the recipient is the fine paid to a regulator for violation of federal privacy rules. There are three notable findings: 1) it is possible to achieve zero risk, in that the recipient never gains from re-identification, while sharing almost as much data as the optimal solution that allows for a small amount of risk; 2) the zero-risk solution enables sharing much more data than a commonly invoked de-identification policy of the U.S. Health Insurance Portability and Accountability Act (HIPAA); and 3) a sensitivity analysis demonstrates these findings are robust to order-of-magnitude changes in player losses and gains. In combination, these findings provide support that such a framework can enable pragmatic policy decisions about de-identified data sharing.

## Introduction

Our ability to collect and analyze personal data has grown dramatically over the past decade, a trend that shows no sign of slowing. While this information enables a wide range of institutions to perform novel research in biomedicine and the social sciences, big data has become big business. There is a rapidly expanding market for sharing and selling data for secondary analysis, driven by profits as well as grant funding agencies aiming to support transparency and research productivity [1, 2]. This movement towards data sharing *en masse* must be accomplished by provisions that appropriately protect the privacy expectations of the individuals to whom the data corresponds [3, 4]. Historically, this has been achieved by removing aspects of an individual’s identity (e.g., suppression of certain demographics), a practice codified in laws around the world, such as the Privacy Rule of the Health Insurance Portability and Accountability Act (HIPAA) [5] and the Data Protection Directive of the European Union (EU) [6]. Such laws provide specific guidance on how to share *de-identified* or *anonymised* information.

The HIPAA Privacy Rule [5] states that only “individually identifiable” information is covered by the regulation. It goes on to state that data is no longer subject to the regulation when it is de-identified. Specifically, this is defined as “information that does not identify an individual and with respect to which there is no reasonable basis to believe that the information can be used to identify an individual is not individually identifiable.” The Privacy Rule then provides alternative implementation specifications, one of which must be followed to ensure that data meets the de-identification definition. The first implementation specification is the Safe Harbor policy, which enumerates 18 attributes that must be generalized or suppressed. The details are provided in Table 1. In this study, we focus on Safe Harbor’s perspective on demographics, which states that 1) all ZIP codes must be rolled back to their initial 3 digits and, further, that codes with populations of less than 20,000 individuals must be grouped into a single code of 000** and 2) ages over 90 must be aggregated into a top-coded age group of 90+.

**Table 1:** The attributes removed, or generalized, to satisfy the HIPAA Safe Harbor policy.

	**Attributes**
(A)	Names
(B)	All geographic subdivisions smaller than a state, including street address, city, county, precinct, ZIP code, and their equivalent geocodes, except for the initial three digits of the ZIP code if, according to the current publicly available data from the Bureau of the Census: (1) The geographic unit formed by combining all ZIP codes with the same three initial digits contains more than 20,000 people; and (2) The initial three digits of a ZIP code for all such geographic units containing 20,000 or fewer people is changed to 000
(C)	All elements of dates (except year) for dates that are directly related to an individual, including birth date, admission date, discharge date, death date, and all ages over 89 and all elements of dates (including year) indicative of such age, except that such ages and elements may be aggregated into a single category of age 90 or older
(D)	Telephone numbers
(E)	Fax numbers
(F)	Email addresses
(G)	Social security numbers
(H)	Medical record numbers
(I)	Health plan beneficiary numbers
(J)	Account numbers
(K)	Certificate/license numbers
(L)	Vehicle identifiers and serial numbers, including license plate numbers
(M)	Device identifiers and serial numbers
(N)	Web Universal Resource Locators (URLs)
(O)	Internet Protocol (IP) addresses
(P)	Biometric identifiers, including finger and voice prints
(Q)	Full-face photographs and any comparable images
(R)	Any other unique identifying number, characteristic, or code, except as permitted by paragraph §164.514(c)

Notice that the policy requires the removal of explicit identifiers (e.g., the names of the corresponding individual or of their relatives, employers, or household members), quasi-identifiers (e.g., attributes that could potentially be linked to identify them, such as demographics), and unique codes (e.g., medical record numbers).

However, a growing collection of investigations demonstrate how de-identified information can be re-identified to the individuals from which it was derived (e.g., via demographics [7–9], genome sequences [10, 11], mobility patterns [12], and social networks [13, 14]). This phenomenon has led to claims that de-identification fails to adequately protect privacy [15, 16]. It has been suggested that society should adopt new definitions of privacy (e.g., [17]) and new *legal* mechanisms to mitigate misuse and abuse of identified personal information (e.g., [18–20]). However, such calls for a revolution are based on demonstrations of what is *possible* and not necessarily what is *probable*. To date, there has been little evidence that such attacks will be realized in practice for any reason other than demonstration [21], and there are many reasons why an adversary may choose to forgo a re-identification attempt in the first place [22]. Rather than viewing de-identification as a dichotomous problem of “broken” or not, it should be considered as a matter of risk over a continuous range.

HIPAA acknowledges this fact by stating in the second implementation specification, which the game theoretic perspective is designed to address, that “information is not individually identifiable health information only if: A person with appropriate knowledge of and experience with generally accepted statistical and scientific principles and methods for rendering information not individually identifiable: (i) Applying such principles and methods, determines that the risk is very small that the information could be used, alone or in combination with other reasonably available information, by an anticipated recipient to identify an individual who is a subject of the information; and (ii) Documents the methods and results of the analysis that justify such determination.” Here, we highlight the fact that the notion of de-identification is explicitly tied to a risk assessment that accounts for the capabilities of a reasonable adversary. We believe this is a clear justification for the application of the game-based approach to the de-identification problem.

Recently, risk-based approaches have been proposed for decision support in de-identification [23]. However, it should be recognized that a key source of re-identification risk is a decision on the part of the data recipient to *attempt* re-identification. It is natural that the anticipated data recipient, in most practical data sharing settings, will only attempt re-identification if there is a tangible economic benefit (e.g., monetary gain) to doing so. Consequently, we model the data recipient as an attacker who will choose to attempt re-identification if the associated expected benefits outweigh the costs. The costs of re-identification can come from numerous sources, including those associated with purchasing data to execute an attack by linking on common features (e.g., residual demographics), as well as the time and resource utilization necessary to run the attack. We rely on this model of a data recipient who is motivated by economic gain as a part of a game theoretic framework that 1) computes the best data sharing strategy for the publisher and 2) accounts for the associated incentives of data recipients to attempt re-identification.

We illustrate our framework through a case study using the demographics reported in a publicly available dataset from the U.S. Census Bureau. In this game, the benefits to the data publisher are proportional to the amount of funding provided to investigators via National Institutes of Health (NIH) grants, and the losses are proportional to fines paid to the U.S. Department of Health and Human Services (HHS) for privacy violations in the form of information security breaches. We begin by computing an optimal data sharing solution in this setting. Interestingly, in this solution the data recipient has the incentive to try to re-identify a significant fraction of records. Nevertheless, the likelihood of a *successful* re-identification is quite low, and the only reason there is any incentive is that it is cheap to attempt it (we set the per-record cost to be only $4, which is roughly the cost for using an online data broker). Our most significant finding, however, is that it is actually possible to achieve *zero* risk, in the sense that the attacker has no incentive to re-identify *any* record in the published data. Remarkably, this zero-risk solution shares nearly as much data as the optimal, but slightly more risky, data sharing policy. Moreover, the zero-risk solution also shares significantly more data than would be shared under the HIPAA-recommended Safe Harbor de-identification policy—even though Safe Harbor also incurs non-zero re-identification risk. Motivated by the ubiquitous use of Safe Harbor as minimal (and, therefore, safe) HIPAA compliance, we additionally consider publishing strategies that satisfy Safe Harbor standards. In this highly restricted setting, we find a policy which, while compliant with Safe Harbor, actually yields a higher utility to the publisher, primarily due to a significantly reduced re-identification risk. Indeed, we observe that these gains are quite substantial for a non-trivial fraction of individuals. Finally, we execute an extensive sensitivity analysis of our findings, and observe that they are robust to order-of-magnitude changes in both gains and losses of the publisher and data recipient.

## Materials and Methods

### Model

Consider an organization (e.g., an academic medical center) that aims to release data with as much fine-grained information as possible, but simultaneously account for the risk of re-identification and concomitant fallout. Re-identification risk has two sources: first, a decision by the data recipient to *attempt* re-identification, aimed at achieving some goal which the publisher views as undesirable (such as imposing a fine on the publisher, or selling re-identified data), and second, the probability that a re-identification attempt succeeds. We model the data recipient as an intelligent attacker who can access external resources at a fixed cost to perform a *linking* attack, where the attributes shared by the data sets are leveraged to connect the corresponding records, and who only attempts re-identification if his associated benefits exceed the costs (which can also include linking and curation costs). If re-identification of a record in the data set is attempted, the probability it succeeds derives from the fact that the external resource could contain the corresponding individual’s identity. For example, de-identified medical records have been linked to voter registration lists to identify individuals and disclose potentially sensitive test results [24].

The ability of the data recipient to successfully re-identify a record hinges on the information released to him by the publisher: the more precise and complete the information, the more likely it would be that a re-identification attack succeeds. In formal notation, we let *g* be the representation of a given record that is released to a recipient, and let *π*(*g*) be the probability of successful re-identification, should it be attempted. The space of data representations we consider involves *attribute generalization hierarchies*, one of the most common paradigms in data de-identification [25]. For example, let us take an individual’s *age*. An age of 22 can be retained in the most specific form, or generalized to a range [20-25], [20-29], [0-50], or * (i.e., any age). In this case, we say the age generalization hierarchy has 5 levels. We assume that such a hierarchy is given for each attribute, so that the publisher’s choice is the level of specificity within it. For attribute *f*, we use *g*_*f*_ and *h*_*f*_ to denote the specific level and the number of levels in the corresponding hierarchy, respectively. In the above example, if the publisher chooses to release the attributes in the most specific form, our representation will be *g*_*f*_ = 0, and *h*_*f*_ = 5. Table 2 summarizes the notation that is useful in this work.

**Table 2:** A summary of the notation used in this work.

**Notation**	**Meaning**
*m*	The number of shared attributes in both released data and external source
*g* = {*g*_1_, … , *g*_*m*_}	The publisher’s strategy on generalization levels for one record
*h*_*f*_	The number of levels in the generalization hierarchy for attribute *f*
*r*	The number of the publisher’s available strategies for one record
*v*(*g*)	The benefit that the publisher receives from sharing data using strategy *g*
*V*	The benefit that the publisher receives by sharing the record in its original form
*L*	The publisher’s loss for one record due to a successful re-identification
*c*	The adversary’s cost to launch a re-identification attack towards one record
*π*(*g*)	The probability the adversary re-identifies one record successfully given strategy
*U*_*p*_(*g*), *U*_*a*_(*g*)	The publisher’s and the adversary’s payoffs given strategy *g*
*a*(*g*)	The adversary’s strategy given strategy *g*
*GI*(*g*)	The generalization intensity of the publisher’s strategy given strategy *g*

If a record is released at specificity *g*, the recipient has two options: either attempt re-identification, incurring a fixed cost *c*, or not. A successful re-identification of a record results in a loss to the publisher which we denote by *L*, and a gain to the attacker (recipient); to keep things simple, we let *L* also be the attacker’s gain, although generalization is direct. If a re-identification fails, or is not attempted, nothing is gained by the data recipient (besides the data, of course) or lost by the publisher. The latter, however, will always obtain a fixed payout *v*(*g*) for a record which is shared at specificity *g*. The expected payoffs of both the publisher and the data recipient (*U*_*p*_ and *U*_*a*_, respectively) are shown in Table 3.

**Table 3:** Payoff functions for the publisher and adversary for a fixed data sharing strategy *g*.

	**no attack**	**attack**
*U*_*p*_(*g*)	*v*(*g*)	*v*(*g*)−*Lπ*(*g*)
*U*_*a*_(*g*)	0	*Lπ*(*g*)−*c*

**Fig 1:**
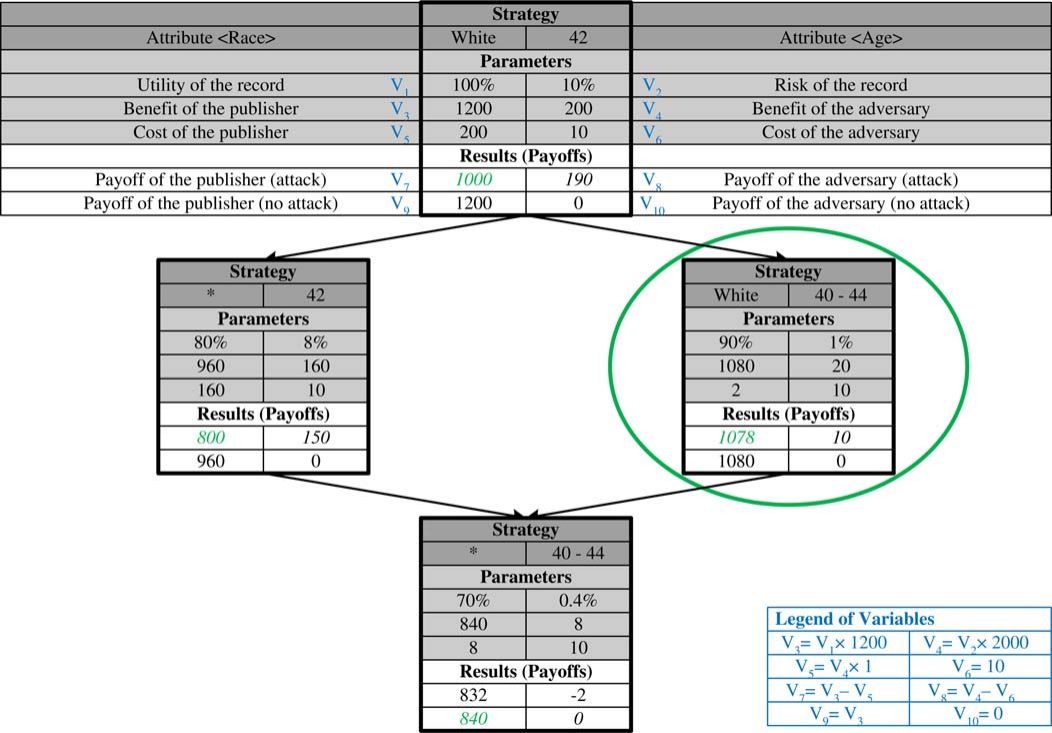
An illustrative example. An illustrative example of the re-identification game with four data sharing strategies.

To make the game concrete, Fig. 1 depicts an example of a simplified version. Here, the publisher has four actions (or strategies) to obfuscate an individual’s record, which correspond to the amount of detail they are willing to reveal about certain personal characteristics. Given each action, the adversary has two responses: i) attack or ii) not. In Fig. 1, each node represents an action of the publisher and each edge represents a partial order relation between two nodes. Both the risk and the utility with the publisher’s action decreases from the top to bottom. To maximize her payoff for each action (marked in green), the publisher’s optimal action is to generalize the age attribute (marked by the green ellipse), while the adversary’s best response is to mount an attack.

In game theoretic terms, the environment we have constructed is a Stackelberg game, in which the publisher first releases the data represented at specificity *g* to the data recipient, who subsequently decides whether or not to attempt re-identification. The solution to this game—a Stackelberg equilibrium—entails a publisher’s decision about an *optimal* representation *g* that maximizes her expected utility over all possible representations, balancing the benefits of releasing as much data as possible (*v*(*g*)) and the risk of re-identification, determined by the data recipient’s decision. We play out this game independently for each record in the data set.

The legal ambiguity of the data de-identification policy landscape warrants several extensions to the basic model above, which we will henceforth call the *Basic Game*. The first is a *No-Attack* game. In this version, we constrain the publisher to choose a representation so that a data recipient driven by economic incentives will never *choose* to attempt re-identification. The second is *SH-Friendly*, in which we constrain representations to be at least as strict as the Safe Harbor (SH) de-identification guideline in HIPAA, so that they could easily explain to an Institutional Review Board (or another authority) why they have selected to share such data. Since both these extensions constrain the set of options available to the publisher, it can be proven they are suboptimal in terms of the underlying cost-benefit tradeoffs the publisher faces, as captured by the model. However, institutional and regulatory oversight may effectively impose these constraints and our analysis provides decision support for data publishers in such environments, as well as a quantitative assessment of the impact such constraints have on the value of de-identified data.

### Solving the Game

We propose two approaches for solving the re-identification game. The first is *backward induction*, in which we consider the adversary’s response, and corresponding player utilities, for each possible choice of publisher’s generalization level, *g*. We then select the generalization level which maximizes the publisher’s utility. Since backward induction requires exhaustive search through the combinatorial space of all feasible representations of data, it clearly would not scale when the number of potentially identifying attributes (often termed *quasi-identifiers*) is large. Consequently, we developed a *lattice-based search* heuristic algorithm which takes advantage of the fact that generalization levels of the attributes form a lattice.

### Backward Induction Search Algorithm

Algorithm 1 in Table 4 reports on the pseudocode for the Backward Induction Search (BIS) method. This is a brute force exploration of the strategy space that guarantees discovery of the optimal strategy. The time complexity of this algorithm is *O*(*r*) = *O*(∏_*f*_ *h*_*f*_). The running time increases linearly with the size of the publisher’s strategy space *r* which is a product of *h*_*f*_ the number of generalization levels for each attribute *f*.

**Table 4:** Algorithm 1: Backward Induction Search (*BIS*) Algorithm.

**Input:** *G* = {*g*}, the set of the publisher’s strategies; *L*, the publisher’s loss; *c*, the adversary’s cost; *W* = {*v*(*g*)}, the set of publisher’s benefits; Π = {*π*(*g*)}, the set of probability of successful attack
**Output:** g*, the publisher’s best strategy
1: *g* ←1st strategy in *G*
2: g* ← *g*
3: **if** *Lπ*(*g*) ≤ *c* **then**
4: *U_m_* ← *v*(*g*)
5: **else**
6: *U_m_* ← *v*(*g*) − *L*π(*g*)
7: **end if**
8: **while** *g.next* ≠ *NULL* **do**
9: *g* ← *g.next*
10: **if** *Lπ*(*g*) ≤ *c* **then**
11: *U_d_* ← *v*(*g*)
12: **else**
13: *U_d_* ← *v*(*g*) − *Lπ*(*g*)
14: **end if**
15: **if** *U_d_* > *U_m_* **then**
16: *U_m_* ← *U_d_*
17: g* ← *g*
18: **end if**
19: **end while**
20: **return** g*

### Lattice-Based Search Algorithm

The BIS approach requires an exhaustive search of all possible strategies. As the number of attributes and the number of generalization levels for each attribute grow, the number of strategies will overwhelm the search making it impossible to search every possible strategy in an efficient manner. As such, we devised a heuristic-driven approach that takes advantage of a natural order to the strategies.

Specifically, the benefit of the publisher *v*(*g*) and the probability of success *π*(*g*) have a partial order on the set of the publisher’s strategies along the generalization level. For instance, imagine strategy *g* ^*i*^ has at least one attribute that is more general than the corresponding attribute of another strategy *g* ^*j*^ while other attributes are in the same generalization level. In this case, the benefit *v*(*g* ^*i*^) and the probability of success *π*(*g* ^*i*^) will never be larger than the benefit *v*(*g* ^*j*^) and probability *π*(*g* ^*j*^), respectively.

As such, the illustration example in Fig. 1 can be regarded as a part of a lattice. The top node of the lattice indicates a strategy sharing all attributes without generalization, while the bottom node of the lattice indicates a strategy of sharing no data.

**Fig 2:**
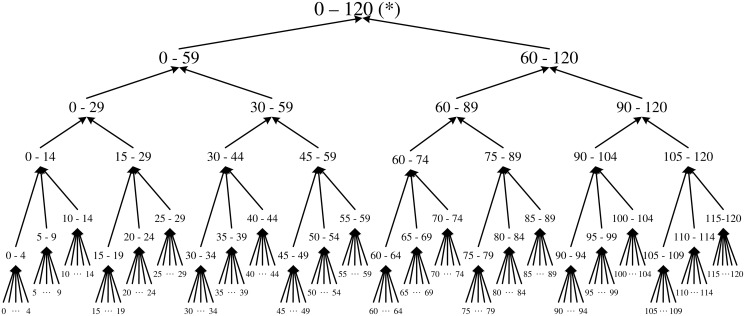
DGH for Age. The Domain Generation Hierarchy (DGH) for the attribute Age in the case study.

**Fig 3:**
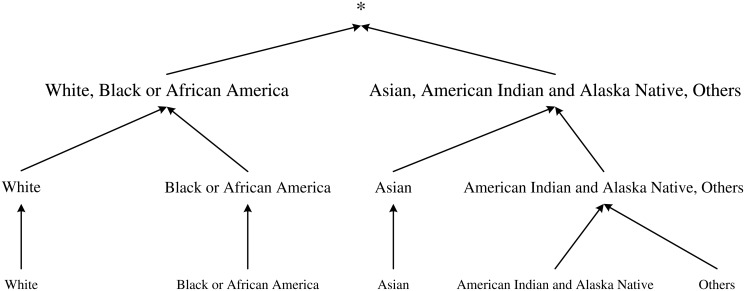
DGH for Race. The Domain Generation Hierarchy (DGH) for the attribute Race in the case study.

**Fig 4:**
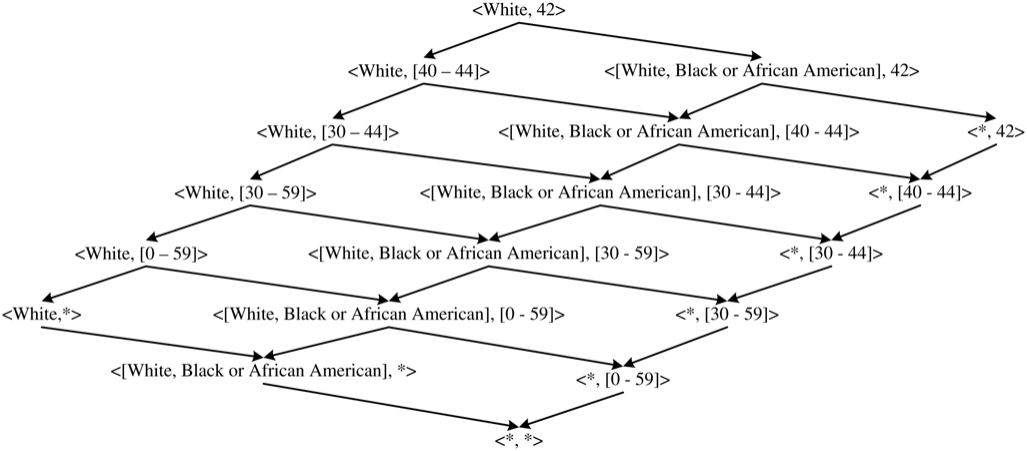
Lattice for two attributes. The illustrative lattice for a database with two attributes Race and Age.

For a database with only two attributes Age and Race, let the domain generalization hierarchy (DGH) for the attribute Age be the one shown in Fig. 2 and the DGH for the attribute Race be the one shown in Fig. 3, then the whole lattice of the strategy space for publishing record ⟨White, 42⟩ has a structure as shown in Fig. 4.

Our lattice-based search (LBS) algorithm is shown in Algorithm 2 in Table 5 and described as follows. The algorithm starts the search from the top node. If *Lπ*(*g*) < *c*, then the adversary will not attack given this strategy nor any strategy represented by any descendent of this node. Thus, all descendants of this node are pruned and the algorithm returns strategy *g*. Otherwise, it searches through every child of the current node and continues with the child with the largest payoff, and prunes the other children. The algorithm halts when either 1) the payoff of the current node is larger than the payoffs of its children or 2) the current node has no children. The time complexity of this algorithm is *O*(*m*) = *O*(∑_*f*_ *h*_*f*_) which is much smaller than the one of backward induction; however, it can not guarantee to find the global optimum. Nevertheless, the algorithm can be improved to find the global optimum by continuing the search among the set of nodes that have not been searched or pruned until the set is empty.

**Table 5:** Algorithm 2: Lattice-Based Search (*LBS*) Algorithm.

**Input:** *G* = {*g*}, the set of strategies for the publisher; *L*, the publisher’s loss; *c*, the adversary’s cost; *W* = {*v*(*g*)}, the set of benefits for the publisher; Π = {*π*{*g*)}, the set of probability of successful attack
**Output:** *g*, the publisher’s best strategy
1: *top* ← [0, 0, …, 0]
2: *g* ← *top*
3: **while** *g.children* ≠ ∅ **do**
4: **if** *Lπ*(*g*) ≤ *c* **then**
5: **return** *g*
6: **end if**
7: *U_m_* ← *v*(*g*) − *Lπ*(*g*)
8: *g_m_* ← *g*
9: **for all** *g_c_* ∈ *g.children* **do**
10: **if** *v*(*g_c_*) − *L*π(*g_c_*) ≥ *U_m_* **then**
11: *g_m_* ← *g_c_*
12: *U_m_* ← *v*(*g_c_*) − *Lπ*(*g_c_*)
13: **end if**
14: **end for**
15: *g* ← *g_m_*
16: **end while**
17: **return** *g*

## Results

### Dataset and Experimental Setup

We evaluated the re-identification game framework in two contexts. First, we compare the data sharing policies computed by the framework to the HIPAA Safe Harbor de-identification policy in a case study. This case study parameterizes the system with evidence-based benefits, costs, and loss values. Second, we perform a sensitivity analysis to understand the relationship between the parameters and the strategies played by the publisher and the adversary.

#### Dataset

Our experiments focus on the Adult dataset, a publicly-accessible extract of the 1994 U.S. Census database [26]. The dataset consists of 48,842 records on 14 personal attributes. For this study, we removed all records with missing values, yielding a dataset of 32,561 records. We use traditional demographics (commonly exploited in re-identification studies) – *Age*, *Race*, and *Gender*, but all ages above 90 were published as one group (90+). As such, we use the publicly available U.S. Census data to disaggregate these ages into years 90 through 120. To compare how changes in geographic features influence identifiability, we added an additional attribute, 5-digit ZIP codes in the state of Tennessee, which was obtained from U.S. Census Bureau’s dataset [27]. We add such data because, according to Safe Harbor, only the first 3 digits of a ZIP code can be disclosed, provided it has at least 20,000 inhabitants. Specifically, for each ⟨Age, Race, Gender⟩ combination, we assign a ZIP code proportionally to the probability distribution reported in the U.S. Census Bureau’s PCT12A through PCT12G tables.

#### Parameters

It should be recognized that the publisher’s benefit is affected by the information loss of the released record. To measure information loss, we define an entropy-based metric *IL* as follows. Given a strategy of the publisher *g* which generalizes an attribute *f* to an interval [*q*_*l*_(*f*, *g*), *q*_*h*_(*f*, *g*)], we assume that *A*_*f*_, the original value of the attribute *f*, is an uniformly distributed random variable in the interval. The probability that *A*_*f*_ equals *q* where *q* ∈ [*q*_*l*_(*f*, *g*), *q*_*h*_(*f*, *g*)] is computed as *P*(*q*, *f*, *g*) = 1/*size*(*q*_*l*_(*f*, *g*), *q*_*h*_(*f*, *g*)).

The total information loss is computed as the sum of the entropy for each attribute as

$$\begin{array}{cc} IL(g) & =\sum_{f}\sum_{q}-P\left(q,f,g\right)\log\left(P\left(q,f,g\right)\right)\\ & =\sum_{f}-\log\left(\frac{1}{size(q_{l}\left(f,g\right),q_{h}\left(f,g\right))}\right) \end{array}$$ (1)

We normalize *IL* by dividing by its maximal value. This corresponds to the scenario where every attribute *A*_*f*_ is generalized to the entire domain *D*_*f*_. The maximal value is computed as

$$\begin{array}{c} Max\left(IL\left(g\right)\right)=\sum_{f}-\log\left(\frac{1}{size(D_{f})}\right) \end{array}$$ (2)

Since the publisher’s benefit *v*(*g*) will decrease as the information loss increases, we estimate it using a linear function in Equation (3), in which *V* corresponds to the benefit that the publisher receives by sharing the record in its original form.

$$\begin{array}{c} v\left(g\right)=V\times \left(1-\frac{IL(g)}{Max(IL(g))}\right) \end{array}$$ (3)

To compute the probability of the adversary’s success *π*(*g*), we adopt the disclosure measure described in [28] which is inversely proportional to the size of population group *n*_*p*_(*g*) that matches the record’s released attribute values.

$$\begin{array}{c} \pi \left(g\right)=1/n_{p}\left(g\right) \end{array}$$ (4)

The cost of the adversary *c* is the price the adversary pays for accessing a record in the external resource used in the linkage attack.

To relate the game to real world de-identification policies, we compare the best strategy of the publisher from the game to the HIPAA Safe Harbor standard. We compare the resulting strategies using the following measures: i) adversary’s payoff *U*_*a*_, ii) publisher’s payoff *U*_*p*_, iii) adversary’s best strategy *a*, iv) generalization intensity of the publisher’s best strategy, and v) the risk of the dataset (e.g., the probability of re-identification, the probability of success if the attack transpires).

We define the generalization intensity *GI*(*g*) as follows:

$$\begin{array}{c} GI\left(g\right)=\frac{\sum_{f}g_{f}}{\sum_{f}h_{f}-m} \end{array}$$ (5)

in which *m* represents the number of attributes. *GI*(*g*) = 0 indicates the publisher’s strategy *g* is to release the record without generalization, and *GI*(*g*) = 1 implies that *g* completely suppresses the record.

### A Case Study in Genomic Data Sharing

In the case study, the publishers are biomedical researchers who are disseminating research datasets. Funding organizations, such as the NIH, require researchers who are granted funding to publish the data generated by their research through websites such as the Database of Genotypes and Phenotypes (dbGaP) [29]. However, while they need to share data, they also have an incentive to protect the identities of the individuals who participated in the original research. The benefit associated with publishing the research dataset can be correlated to the amount of funding received for the project. For example, consider the dataset [30] in dbGaP submitted by the five separate member institutions of the NIH-sponsored Electronic Medical Records and Genomics Network (EMERGE) [31]. According to the NIH [32], the total sum of grant funding provided for the project is $22,272,084. Since there are 18,663 entries, we estimate that the publisher’s benefit associated with each record is *V* = $22,272,084/18,663 ≈ $1,200.

To the best of our knowledge, there has never been a fine levied for the re-identification of data. Thus, as a proxy, we assume the *loss* of the publisher for each re-identified record *L* could be proportional to the fine paid to a federal regulator for a data breach as reported on the Office for Civil Rights’ *Wall of Shame* on the U.S. Department of Health and Human Services (HHS)’s website [33]. As such, we set *L* = $300 according to the average fines per record, which is $325.95, recognizing that the statutory penalties could be much higher. Table 6 provides a summary of the fines and number of records involved in recent HIPAA violation breach cases. The sensitivity analysis in the following section provides intuition into how other types of loss, such as that which is incurred through identity theft blackmail, influences the results of the game.

**Table 6:** Recent notable HIPAA breach violation cases as reported by the U.S. Department of Health and Human Services.

**Entity**	**Fine**	**#Records**	**Fine/record**	**Date**
New York and Presbyterian Hospital	$4,800,000	6,800	$705.9	May 7, 2014
QCA Health Plan, Inc.	$250,000	148	$1689.2	Apr. 22, 2014
Skagit County, Washington	$215,000	118,000	$1.8	Mar. 7, 2014
Adult and Pediatric Dermatology	$150,000	2,200	$68.2	Dec. 26, 2013
Affinity Health Plan, Inc.	$1,215,780	344,579	$3.5	Aug. 14, 2013
WellPoint, Inc.	$1,700,000	612,402	$2.8	Jul. 11, 2013
Idaho State University	$400,000	17,500	$22.9	May 21, 2013
The Hospice of North Idaho	$50,000	441	$113.4	Jan. 2, 2013

We set the cost of the adversary *c* for each external identified record to $4 (based on the $3.95 price for a basic report from www.intelius.com).

Finally, we set the number of generalization levels *h* for each of the attributes Age, Race, Gender, and Zip to be 6, 4, 2, and 6, respectively, to ensure the publisher has a relatively large strategy space (a space of 288 strategies), considering the number of distinct values for these attributes are 121, 5, 2, and 628, respectively. We construct the generalization hierarchies such that i) HIPAA Safe Harbor de-identification policy is always a strategy in the publisher’s strategy space; ii) for Age, Race, and Gender, each level has a relatively certain number of distinct values (e.g., the number of distinct values in 6 levels of the attribute Age from top to bottom is 121, 60 or 61, 30 or 31, 15 or 16, 5 or 6, and 1, respectively); iii) for Zip, each level has a distinct form of representation (e.g., for Zip 37203, the 6 levels from top to bottom are represented as *****, 3****, 37***, 372**, 3720*, and 37203, respectively) except Zip 42223, 42602, and 72338 which has fewer than 20,000 residents (e.g., for Zip 42223, the 6 levels from top to bottom is represented as *****, [4****, 7****], [42***, 72***], [422**, 426**, 723**], 4222*, and 42223, respectively).

**Fig 5:**
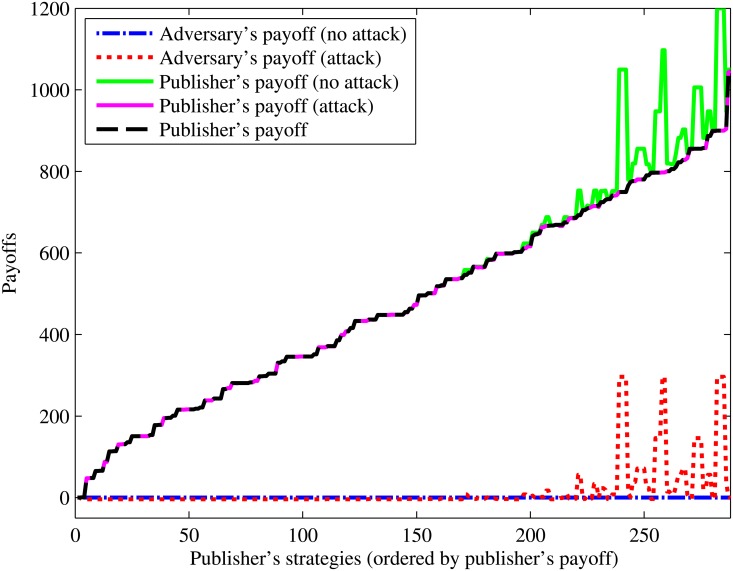
Payoff across strategies. Payoffs for the record ⟨48, Asian, Female, 38363⟩ across all strategies.

For orientation, Fig. 5 demonstrates the publisher’s and adversary’s payoffs across all strategy profiles for the record ⟨48, Asian, Female, 38363⟩ in the *Basic Game*. In this case, the publisher’s best strategy is to release the record as ⟨48, *, *, 38363⟩. The resulting generalization intensity is 0.29 and the adversary’s probability of success is 0.0104. The adversary’s best response is *no attack*, such that his payoff is $0 while the publisher’s payoff is $1,049.70. Now, if the publisher had invoked the HIPAA Safe Harbor policy, the record would have been released as ⟨48, Asian, Female, 383**⟩, which yields a generalization intensity of 0.14 (less intensive) and the adversary’s probability of success would have been 0.1429, which is approximately 14 times of the rate of the *Basic Game*. Moreover, unlike the *Basic Game*, the adversary’s best response is to *attack* this record. As a consequence, the adversary’s expected payoff increases to $38.86, and the publisher’s payoff decreases to $776.30. For this record, the best strategy from the *Basic Game* outperforms the Safe Harbor policy.

Turning our attention to the entire dataset, Table 7 summarizes the results of all variations of the re-identification game and Safe Harbor on several measures. In the *Basic Game*, it can be seen that the average payoff to the publisher and the adversary for each record is $1,195.50 and $2.43, respectively. For every record, the publisher’s best strategy is to share data. In 62.98% of the time, records are shared in their most specific form. At the same time, the adversary’s best strategy is to attack 21.87%, or 7122, of the records. However, the average probability of a successful re-identification for any record is 0.0110. And, for each of the records attacked, the average probability of success is 0.0504, such that the expected number of re-identified records would be ∼ 359.

**Table 7:** A comparison of four de-identification policies for the case study on performance measures.

**Performance Measures**	**SH**	**Basic**	**SH-Friendly**	**No-Attack**
Publisher’s average payoff over all records	$852.06	$1,195.50	$852.51	$1,169.80
Adversary’s average payoff over all records	$0.43	$2.43	$0.05	0
Proportion of records with *GI* = 1 (suppression)	0	0	0	0
Proportion of records with *GI* = 0 (most specific)	0	62.98%	0	62.73%
Average GI over all records	0.1431	0.0280	0.1453	0.0412
Proportion of records the adversary will attack	2.68%	21.87%	0.91%	0
Average probability of re-id over all records	0.0110	0.0018	0.0003	0
Average probability of re-id over all attacked records	0.0504	0.0668	0.0310	0

SH: Safe Harbor. GI: Generalization Intensity.

Under the Safe Harbor policy, the average payoff to the publisher and adversary for each record is $869.84 and $0.43, respectively. In this scenario, the adversary’s best strategy is to attack 2.68%, or 873, of the records. The average probability of a successful re-identification for any record is 0.0018. For each of the attacked records, the average probability of success is 0.0668, such that the expected number of re-identified records is ∼ 58.

In the *No-Attack Game*, it can be seen that the average payoff for the publisher is $1,169.80 (while, of course, the adversary receives a payoff of $0). In this situation, the publisher’s best strategy is to share all the records, while 62.73% of the records are shared without any generalization.

In the *SH-Friendly Game*, the average payoff to the publisher and adversary for each record is $852.51 and $0.05, respectively. The publisher’s best strategy is to share all records with generalization, while the adversary’s best strategy is to attack 0.91%, or 295, of the records. The average probability of a successful re-identification for any record is 0.0003. For each of the attacked records, the average probability of success is 0.0310, such that the expected number of re-identified records is ∼ 9. The best strategies from the *No-Attack Game* and *SH-Friendly Game* both unambiguously outperform the Safe Harbor policy by achieving higher payoffs for the publisher as well as lower payoffs for the adversary.

**Fig 6:**
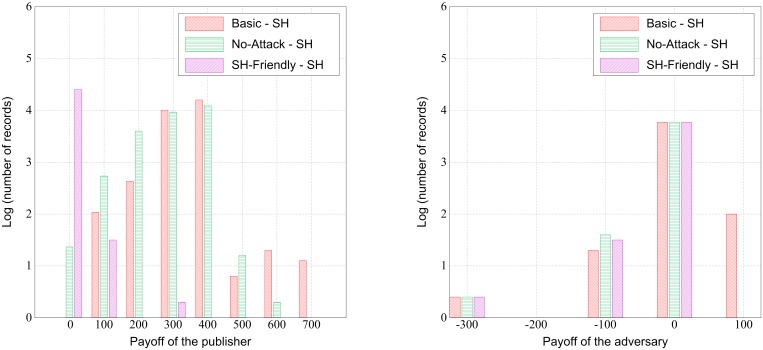
Histogram of Payoff Differences. Distributions of the publisher’s payoff differences (left) and the adversary’s payoff differences (right) between games and HIPAA Safe Harbor (SH).

To investigate these results in greater depth, Fig. 6 depicts the distribution of payoff gains for the *Basic*, *SH-Friendly*, and *No-Attack* games, relative to the HIPAA Safe Harbor policy (in other words, how much better each of these performs than Safe Harbor from the publisher’s and adversary’s perspectives). In Fig. 6, the plot to the left displays the frequency distribution of payoff differences for the publisher. It can be observed that the publisher almost always receives a higher payoff using our framework. Among the three games, the *Basic Game* is the best and the *SH-Friendly Game* is the worst. The plot to the right displays the frequency distribution of payoffs for the adversary. Here, it can be observed that, at times, the adversary receives a higher payoff only via the *Basic Game*. This is because the publisher optimizes the payoff regardless of the risk and, in certain instances, the risk increases in tandem with the payoff.

**Fig 7:**
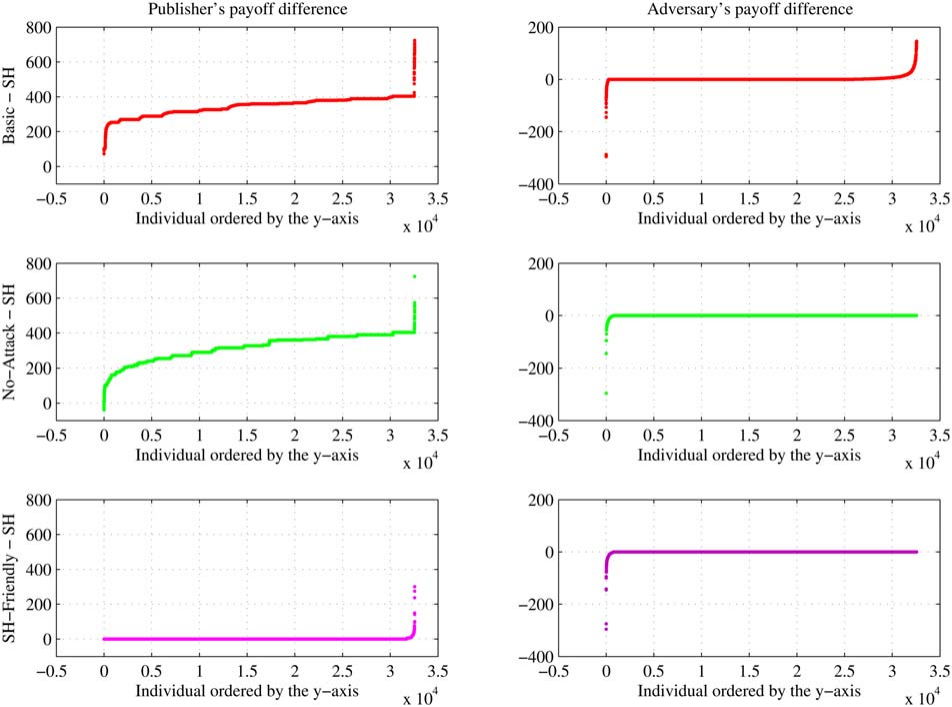
Scatter-plot of Payoff Differences. Detailed distributions of the publisher’s payoff differences (left) and the adversary’s payoff differences (right) between games and HIPAA Safe Harbor (SH).

Fig. 7 depicts the detailed distributions of the publisher’s and the adversary’s payoffs between the games and the HIPAA Safe Harbor de-identification policy for all individuals. First, we consider the viewpoint of the publisher. In the plots on the left, it can be observed that only the *No-Attack Game* has a smaller payoff than Safe Harbor for the publisher. And, among the variations on the de-identification game, the *Basic Game* has largest publisher’s payoff. Next, we turn our attention to the adversary. In the plots to the right, it can be observed that only the *Basic Game* has the capability of achieving a larger payoff than Safe Harbor for the adversary. Among the three games, the *SH-Friendly Game* exhibits the smallest payoff for the adversary.

### Sensitivity Analysis of the Game

In the re-identification game, the Stackelberg equilibrium will remain unchanged if the parameters *L*, *V*, *c* are multiplied by a constant factor. As such, we vary two parameters while holding the third constant.

**Fig 8:**
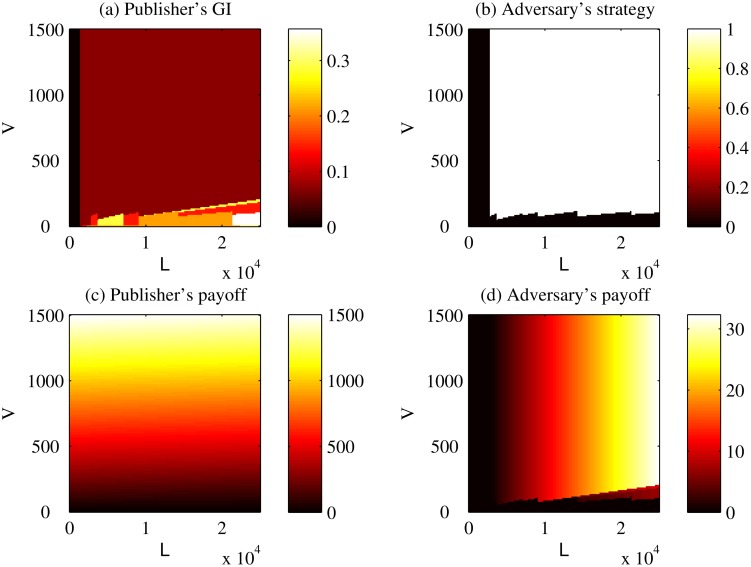
Example solution 1 in Basic Game. Resulting strategies and payoffs for ⟨19, Black or African American, Male, 37208⟩ in the *Basic Game*. GI stands for the generalization intensity. *V* corresponds to the benefit that the publisher receives by sharing the record in its original form. *L* corresponds to the publisher’s loss for one record due to a successful re-identification.

**Fig 9:**
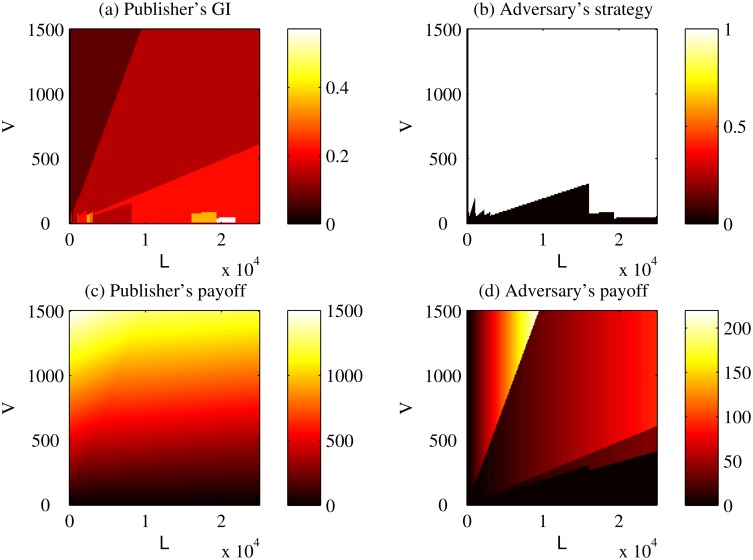
Example solution 2 in Basic Game. Resulting strategies and payoffs for ⟨62, White, Female, 37014⟩ in the *Basic Game*. GI stands for the generalization intensity. *V* corresponds to the benefit that the publisher receives by sharing the record in its original form. *L* corresponds to the publisher’s loss for one record due to a successful re-identification.

Figs. 8 and 9 show the best strategy for the publisher (in the form of GI) and the adversary for two records with vastly different adopted strategies. Specifically, Figs. 8 and 9 correspond to ⟨19, Black or African American, Male, 37208⟩ and ⟨62, White, Female, 37014⟩, respectively.

For orientation, in Fig. 8(a), a GI of 1 (white at the top of the color bar) implies that the publisher will not release anything; a GI of 0 (black at the bottom of the color bar) implies that the publisher will release everything; and a GI between 0 and 1 implies that the publisher will generalize part of the record. From Fig. 8(a), it can be observed that the whole space is separated into several parts that within each of them the publisher’s GI keeps the same. Generally, The color of the part representing high *V* and low *L* is darker than that of the part representing low *V* and high *L*. However, it is not necessary true that the publisher uses a larger amount of generalization for the record when *L* is high and *V* is low. In Fig. 8(b), the white and black areas indicate that the adversary chooses to attack and not attack, respectively. From Fig. 8(b), it can be observed that a frontier cuts the whole space into two parts represent different strategies for the adversary. The adversary will not attack when *L* or *V* is small. In Fig. 8(c), the brighter the region, the larger the publisher’s payoff. In Fig. 8(c), clearly the publisher’s payoff increases as *V* increases. In Fig. 8(d), the brighter the region, the larger the adversary’s payoff. From Fig. 8(d), it can be observed that the whole space is separated into several parts that within each of them the adversary’s payoff increases only as *L* increases. The pattern of separation in Fig. 8(d) is the same as that in Fig. 8(a).

Similar results can be observed in Fig. 9. The differences between Fig. 8 and Fig. 9 include apparently different patterns of separation for the publisher’s GI and different frontiers for the adversary’s strategy.

For these two records, we find that both the publisher’s strategy and the adversary’s strategy are not sensitive to the changes of *V* and *L* near the point representing the case study (*V* = $1,200, *L* = $300, *c* = $4).

**Fig 10:**
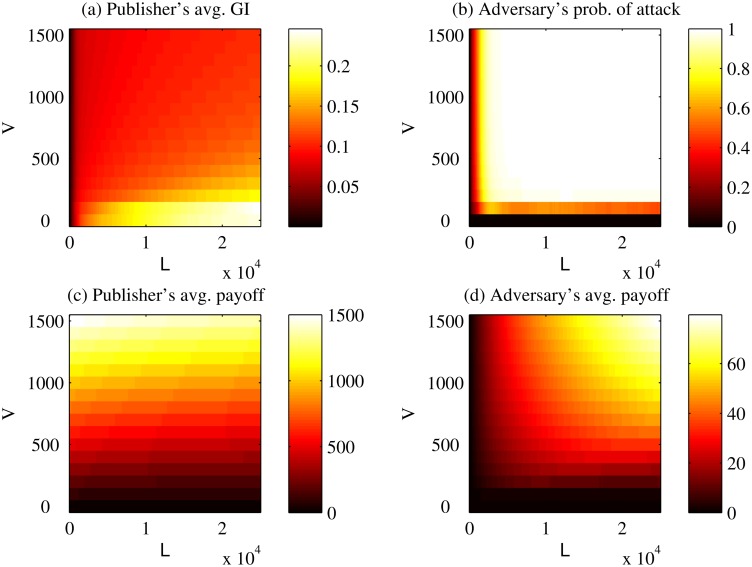
Sensitivity of Basic Game to Benefit and Loss. Sensitivity of the average payoffs and strategies over the dataset to the changes of the publisher’s benefit and the publisher’s loss in the *Basic Game*. GI stands for the generalization intensity. *V* corresponds to the benefit that the publisher receives by sharing the record in its original form. *L* corresponds to the publisher’s loss for one record due to a successful re-identification.

Fig. 10 depicts the publisher’s GI, adversary’s strategy and both payoffs for the entire dataset while varying *L* and *V* in the *Basic Game*. In Fig. 10(a), the brighter the region, the larger the publisher’s average GI. It can be observed that the publisher, on average, generalizes less intensively (i.e., shares more data) than the Safe Harbor policy (0.1431) when either *L* ≤ $4,200, or *V* ≥ $100; thus, this general finding holds over an order-of-magnitude range of both of these parameters. In Fig. 10(b), the brighter the region, the larger the chance the adversary will attack. We can see that the adversary will attack fewer than 30% of the records when either *L* ≤ $1,000 (giving us an order-of-magnitude range of robustness for *L*, for any value of *V* up to $1,500) or *V* ≤ $100 (independent of *L* up to $25,000). In Fig. 10(c), the brighter the region, the larger the publisher’s average payoff. As expected, the publisher’s payoff is correlated with the publisher’s benefit *V*, such that when the benefit is large, the payoff is large as well. In Fig. 10(d), the brighter the region, the larger the adversary’s average payoff. The adversary’s payoff is large for instances where the publisher’s benefit and loss are large.

**Fig 11:**
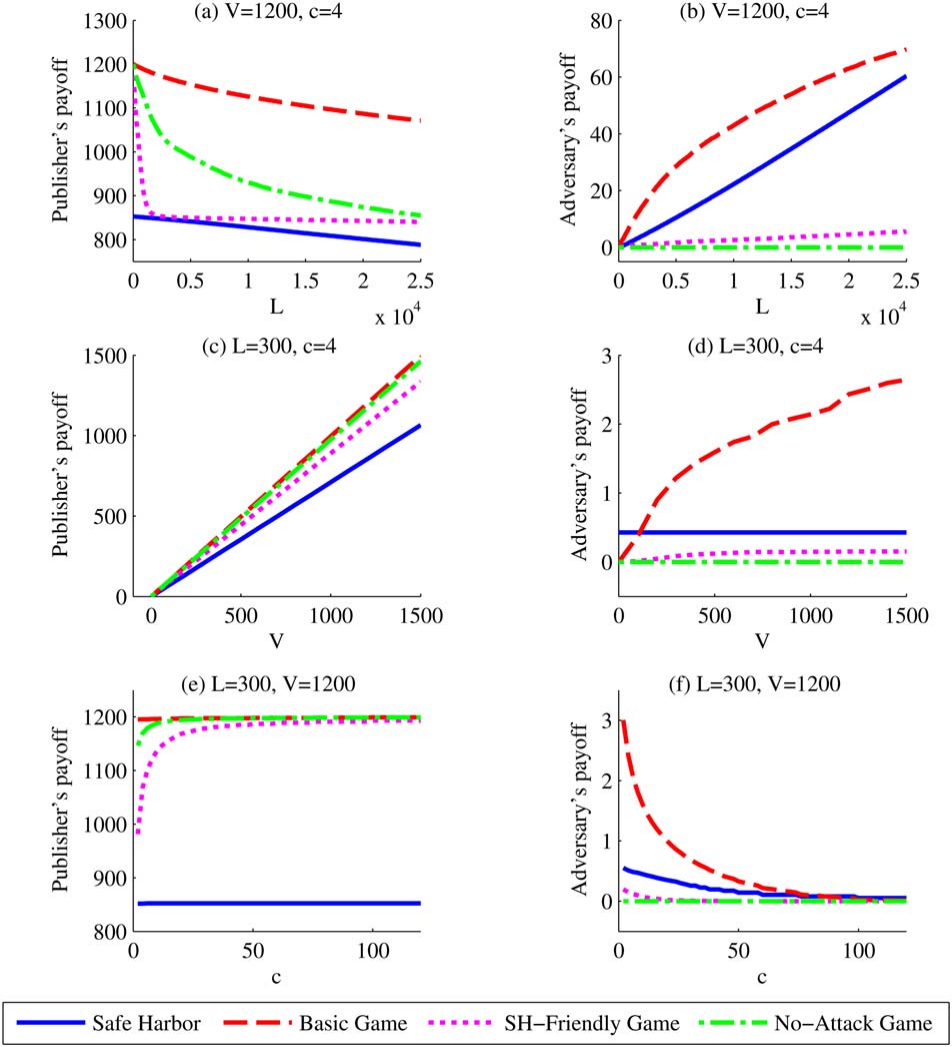
Sensitivities of different scenarios to Benefit or Loss. Sensitivity of the average payoffs over the dataset to the change of the publisher’s benefit, the publisher’s loss, or the adversary’s cost in different de-identification scenarios. *V* corresponds to the benefit that the publisher receives by sharing the record in its original form. *L* corresponds to the publisher’s loss for one record due to a successful re-identification. *c* corresponds to the adversary’s cost to launch a re-identification attack towards one record.

In Fig. 11, we compare the publisher’s and adversary’s payoffs for the Safe Harbor policy with the *Basic*, *No-Attack*, and *SH-Friendly* games by changing *L*, *V*, or *c* only. It can be observed from Fig. 11(a), Fig. 11(c), and Fig. 11(e) that all three games lead to better payoffs for the publisher comparing to Safe Harbor. In addition, Fig. 11(b), Fig. 11(d), and Fig. 11(f) illustrate that the *No-Attack* and *SH-Friendly* games protect the data better than Safe Harbor does. Moreover, when *V* is smaller than $200, or *c* is larger than $90, the *Basic Game* outperforms Safe Harbor as well. From Fig. 11, it can be observed that the increase of *L* benefits the adversary only; the increase of *c* benefits the publisher only; and the increase of *V* benefits both, though more for the publisher.

### Performance Comparison of Game Solvers

In this subsection, we compare the performance of the BIS and LBS algorithms to determine if 1) the latter is more efficient, and 2) the results of LBS are sufficiently close to the baseline BIS. To do so, we rely upon two categories of evaluation metrics. The first category corresponds to quality measures, which include, i) the publisher’s average payoff, ii) the adversary’s average payoff, iii) the average absolute difference of the publisher’s payoff *D*_*p*_ between the two algorithms, and iv) the average absolute difference of the adversary’s payoff *D*_*a*_ between the two algorithms. The average absolute difference of the publisher’s payoff is calculated as follows. Let the publisher’s payoff using BIS and LBS for record *i* be *U*_*BIS*_(*i*) and *U*_*LBS*_(*i*), respectively, and *n* be the total number of the records. Then, the average absolute difference of the publisher’s payoff is calculated as

$$\begin{array}{c} D_{p}=\frac{\sum_{i=1}^{n}\left|U_{BIS}\left(i\right)-U_{LBS}\left(i\right)\right|}{n}. \end{array}$$ (6)

For instance, if the publisher’s payoffs are $700, $800, and $900 in the results of the baseline BIS, and $700, $805, $899 in the results of a heuristic-driven LBS, then the publisher’s average payoff will be $801 and the average absolute difference of the publisher’s payoff is $2. The average absolute difference of the adversary’s payoff is calculated similarly.

The second category of measures correspond to efficiency of the strategy search process: v) the average running time and vi) the average number of strategies (or nodes in the lattice) searched. We retain the same parameter settings introduced in the previous section.

Table 8 provides a summary of the comparison results for BIS and LBS. To measure the runtime (in milliseconds, or ms), we use a Intel CORE i5 2.67GHz machine, with 4GB RAM. We apply a two-sample t-test at the 5% significance level to show that the running times of BIS and LBS are significantly different. It is clear that the LBS approach runs faster than the baseline approach (BIS). The LBS approach ends up with almost the same average payoff for the publisher, and even less average payoff for the adversary. Additionally, 99.47% of the solutions returned from LBS were the optimal solutions.

**Table 8:** A performance comparison of the de-identification game solving approaches.

**Metric**	**BIS**	**LBS**
Running time (ms)	268.1	15.8
Searched nodes	704	3.9
Publisher’s Average Payoff	$1195.5	$1195.2
Adversary’s Average Payoff	$2.43	$0.12
Publisher’s Average Payoff Difference	0	$0.29
Adversary’s Average Payoff Difference	0	$2.64

BIS: Backward Induction Search. LBS: Lattice-Based Search. Payoff difference means the absolute difference of payoff for one record between a heuristic-driven approach and the baseline BIS approach.

**Fig 12:**
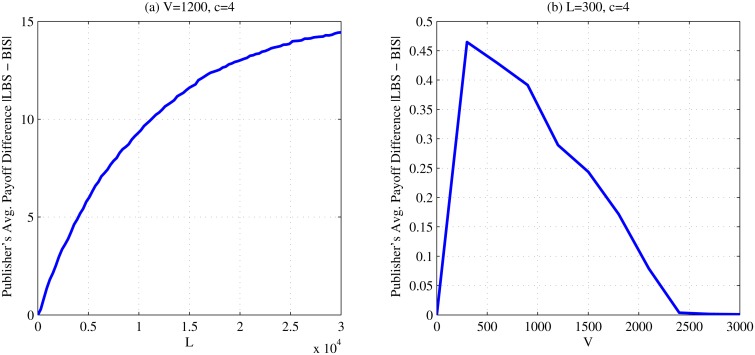
Accuracy of Lattice-based Search. A comparison of the accuracy of the LBS game solving heuristic. *V* corresponds to the benefit that the publisher receives by sharing the record in its original form. *L* corresponds to the publisher’s loss for one record due to a successful re-identification. *c* corresponds to the adversary’s cost to launch a re-identification attack towards one record.

Next, to illustrate the sensitivity of quality metrics with respect to *L* and *V*, we plot the the average absolute deviation of the publisher’s payoff *D*_*p*_ from the baseline solution in Fig. 12. From Fig. 12(a), we observe that, when *V* = $1,200, *c* = $4, as *L* increases, the LBS solution deviates from the BIS baseline, but with a weakening trend. From Fig. 12(b), when *L* = $300, *c* = $4, the LBS solution deviates most when *V* is around $300 and then converges to BIS as *V* increases.

## Discussion

The results of this study are notable for several reasons. First, they illustrate a new formal framework for analyzing data de-identification risk which builds on game theory in order to capture the motivations of a data recipient for re-identifying such data. This framework enables us to identify and rigorously support a de-identification policy which optimally trades off the value of shared data and the associated re-identification risk. Second, our results demonstrate that it is actually possible to achieve zero risk, in the sense that a data recipient would have no incentive to attempt re-identification (with limitations discussed below). Remarkably, this “zero-risk” policy shares nearly as much data as an optimal policy and far more data than the popular HIPAA Safe Harbor de-identification policy. Third, even if we impose Safe Harbor guidelines as a strict constraint on the kinds of de-identification policies we can consider, our results demonstrate that a number of individuals are served poorly by Safe Harbor, in the sense that it releases *too much* information about them. Our model significantly improves outcomes by clamping down on the information release for these individuals.

The legal and institutional complexity of data sharing risk, as well as uncertainty of enforcement guidelines, make rigorous risk analysis for data sharing particularly challenging. Our general framework, as well as a case study illustration, suggest that a formal approach based on a game theoretic model is defensible in the light of regulatory oversight (it is motivated by, and compliant with, the HIPAA notion of an anticipated data recipient) and sufficiently flexible to offer data publishers real alternatives to balance their data sharing needs with institutional and legal requirements, as well as their own risk preferences.

### Limitations

This study has several limitations that can serve as guideposts for future research. The first limitation is that our case study assumed a single source of side data. Thus, the results could potentially change if other related side data can be utilized. However, we emphasize that this is not a limitation of our model, which captures arbitrary side data (the cost and probability of re-identification would then correspond to the most efficacious re-identification policy). Second, our study imposes a fixed generalization hierarchy for all records, thereby significantly limiting the granularity of publisher’s decision space. Our framework does not readily admit adding noise to published data release, and this is an important problem to consider in future work. The third limitation is that our model assumes a single adversary (data recipient). In future work it would be desirable to generalize the model to capture the scenario of multiple data recipients, the uncertainty about the payoffs and information of data recipients, as well as the possible constraint that the same representation of data is shared with all recipients, which would give rise to a multi-follower Bayesian Stackelberg game as the one in [34] and [35]. When multiple types of adversaries are involved, cooperation and competition among them may make the game similar to the prisoner’s dilemma game or the public goods game, for which there has been recent research in solving the problem in a scalable fashion [36, 37]. The fourth limitation is that the model makes no provision for other sources of data re-identification risk, such as another party breaking into a data recipient’s systems and stealing the data. This third party may well have strong non-economic motivations to re-identify data. The clear implication is that our measure of risk (including settings with “zero” risk) underestimates true risk of re-identification. Capturing this background risk of data sharing is also an important subject for future work.

## Conclusions

De-identification has been a hallmark of data protection for years, but there is a fear that this technique is insufficient given mounting re-identification evidence. However, before we throw out de-identification in favor of other technical and legal mechanisms, it should be recognized that it is fundamentally a problem of risk management and that data will be compromised only if adversaries are sufficiently incentivized to do so. To enable pragmatic discussion about de-identification and potential concerns, we introduced a novel formalization of the re-identification problem as a Stackelberg game between a data publisher and recipient. We translated the risk and the utility of the released dataset into the data publisher’s benefit and cost, and proposed several methods for computing the optimal data sharing policy for the publisher. We illustrated our model using a real case study, showing that we can typically achieve much better outcomes for the publisher than HIPAA Safe Harbor, a popular real-world de-identification policy, often at lower re-identification risk. Indeed, our results indicate that publishers can choose strategies that allow for sharing a significant amount of data (far more than under Safe Harbor) while ensuring that it is never beneficial for the data recipient to attempt re-identification, thus ensuring zero risk within the context of our modeling framework.

## Supporting Information

S1 Supporting Information Details regarding privacy risks and data protection models. Details regarding de-identification and anonymization models, the different ways in which re-identification risk are formalized and quantified, and other related investigations into game theory for characterizing and addressing privacy and security concerns.
(PDF)
