# Supplementary material for: A Game Theoretic Framework for Analyzing Re-Identification Risk
Source: PLoS One. 2015 Mar 25;10(3):e0120592. doi: 10.1371/journal.pone.0120592 (PMC4373733; doi:10.1371/journal.pone.0120592)
Supplement: S1 Supporting Information — Details regarding de-identification and anonymization models, the different ways in which re-identification risk are formalized and quantified, and other related investigations into game theory for characterizing and addressing privacy and security concerns. (PDF) [file pone.0120592.s001.pdf]

A Game Theoretic Framework for Analyzing Re-identification Risk : Supporting Information

Zhiyu Wan[1], Yevgeniy Vorobeychik[1], Weiyi Xia[1], Ellen Wright Clayton[2], Murat Kantarcioglu[3], Ranjit Ganta[3], Raymond Heatherly[4], Bradley A. Malin[4,*]
1 Department of Electrical Engineering and Computer Science, Vanderbilt University, Nashville, Tennessee, United States of America
2 Center for Biomedical Ethics and Society, Vanderbilt University, Tennessee, United States of America
3 Department of Computer Science, University of Texas at Dallas, Richardson, Texas, United States of America
4 Department of Biomedical Informatics, Vanderbilt University, Nashville, Tennessee, United States of America
∗ E-mail: b.malin@vanderbilt.edu (BAM)

Abstract

In this Supporting Information appendix, we provide additional details regarding privacy risks and data protection models. First, we review de-identification and anonymization models. In doing so, we provide justification for the generalization strategy invoked in the data protection strategy studied in our work. Second, we recount the different ways in which re-identification risk are formalized and quantified. Third, we contextualize this research with respect to other investigations into game theory for characterizing and addressing privacy and security concerns.

De-identification Models

In the re-identification game studied in this article, we assume the defender's strategy set is derived from de-identification models. Various models have been developed, but all aim to transform the attributes that could be used to ascertain an individual's identity to address *identity disclosure* risk. While there are other privacy concerns (e.g., attribute disclosure [1], presence / absence in a dataset [2], and contribution to a statistical distribution [3]), we focus on identity disclosure because of its direct relationship with existing privacy laws and a broad class of data protection methodologies [4]. We believe that our game theoretic framework will generalize to other privacy models, such as differential privacy [3] which applies random noise to shared data, but focus on identity disclosure for illustration of the novel perspective games bring to the de-identification problem.

The operations that can be applied to de-identify a record can grossly be characterized as i) randomization, ii) generalization, and iii) suppression. We focus on generalization and suppression because they are widely adopted in data protection policies and de-identification algorithms. De-identification policies, such as HIPAA's Safe Harbor [5], often use rules, in the form of an enumerated list of features that need to be generalized (e.g., 5-digit Zip code needs to be generalized to first 3-digits, provided there are at least 20,000 people in the region). Similar rule-based policies have been invoked in other countries, such as Canada [6]. These policies are not necessarily *optimal*, per se, and so de-identification policy search methods have been proposed to discover policies that maximize data utility while satisfying a risk threshold [7–9].

Beyond rule-based policies, other approaches have focused on ensuring the dataset itself satisfies a certain level of protection. For instance, k-anonymity [10] states that a record must be equal to k-1 other records. While k-anonymity can be achieved through any of the aforementioned operations [11], the most common approach is generalization [12]. We adopt the generalization model without enforcing a specific protection parameter. Rather, we search for a generalization that maximizes the payoff for the publisher of a record.

A number of generalization models have been developed (particularly with application for k-anonymization) and it is important to clarify which is used in this work. Specifically, we use a *full-domain generalization* model [13], which is the cross-classification of the domain generalization hierarchy (DGH) for each attribute. This is the most frequently used in practice, but note our framework can be extended for other generalization models, such as full-subtree generalization [14] and multi-dimensional generalization [15].

Risk of Privacy Violations

No system is impregnable to attack and, thus, re-identification risk assessments must be performed. [16] suggested three models of re-identification risks: i) prosecutor, ii) journalist, and iii) marketer. For these risks, it is assumed there is a published dataset, which is based on a sample of a broader population. The prosecutor and journalist risks correspond to the most re-identifiable record in the dataset and population, respectively. The marketer risk, by contrast, corresponds to the average risk of all records in the dataset. While [17] provides mathematical definitions of these scenarios, it is assumed that the attack will always be attempted. However, as we show in this work, the cost of a privacy violation (e.g., expected loss in terms of a fine) greatly influences this decision. There have been investigations into the cost of privacy violations. For instance, [18] introduced quantitative methods to define privacy violations and their consequences. They provided definitions of sensitivity and severity (of privacy violations), taking into account the level at which the data subjects are concerned with regards to their own privacy. [19] designed a decision theoretic framework to assess privacy risk that accounts for both the entity identification and the disclosed information sensitivity. However, these models did not consider multiple players in a game with varying strategies.

One of the challenges associated with re-identification is that an adversary must obtain a degree of *background* knowledge in order to perpetrate their attack. In certain instances, this knowledge may be gained by observation, such as when the adversary sees an ambulance leaving their neighbor's house. Yet such information may be difficult to come by and, thus, it has been suggested that reasonable adversaries are more likely to use resources, such as public records or information brokers, that can be gathered or queried *en masse*. In this regard, there has been some investigation into the credentials and costs associated with gathering such resources. In particular, [20] illustrated that voter registration records, which have been used for re-identifications, have a wide range in price (from \$0 to \$17,000), which is set by the state or municipality making them available, and the amount of information useful for re-identification (e.g., demographics) is not correlated with the price (e.g., the most expensive resource actually had the least amount of information).

Games Applied to Privacy and Security

Game theoretic frameworks have been introduced to model privacy and security problems. [21] proposed a security game model between the defender (e.g., police officers) and the adversary (e.g., terrorists) to optimize the allocation of limited security resources, which is extended in [22] by considering the surveillance cost and partial knowledge of the adversary. In [23], the authors defined a multi-party game to formulate a privacy-preserving distributed data mining problem. However, in this game, every party is both a data publisher and adversary, which is different from our two-player game. In [24], a normal form game between a user and a service provider was defined for assessing privacy risk. In this setting, the user chooses whether or not to provide private information, while the service provider chooses whether or not to exploit the user's private information. Their strategy set is significantly smaller than the one we consider. [25] modeled the location privacy protection problem as a two-player, zero-sum, signaling game. However, the sum of the payoffs of two players in our game model is not zero.

The Stackelberg game model, which we leverage to model the re-identification game, has been used in various contexts. [26], for instance, modeled the adversarial prediction problem as a Stackelberg game

between a data generator (leader) and a learner (follower). Here, the leader generates data based on the follower's prediction models to create confusion, while the follower adjusts the prediction models to account for the leader's response. [27] modeled the adversarial prediction problem as a single-shot game, which is one kind of Stackelberg game as we used in our model, and explored the conditions for the existence of unique Nash equilibrium. [28] introduced the notion of games for auditing the use of medical records in the context of primary care settings and presented a polynomial-time approximation scheme to compute a solution that is arbitrarily close to the optimal solution. Their approach to computing the Stackelberg equilibrium is based on the multiple-LPs technique of Conitzer and Sandhom [29]. In their Stackelberg game model, the defender is the data publisher (e.g., hospital) and the adversary is the data recipient (e.g., employee), which is very similar to the settings in our game model. However, while they can only mitigate the privacy risk after the data publishing, we minimize the risk even before the data publishing.

References

1. Machanavajjhala A, Kifer D, Gehrke J, Venkitasubramaniam M (2007) l-diversity: Privacy beyond k-anonymity. ACM Trans on Knowledge Discovery in Data 1.

2. Nergiz ME, Atzori M, Clifton C (2007) Hiding the presence of individuals from shared databases. In: Proceedings of the ACM SIGMOD International Conference on Management of Data. pp. 665–676.

3. Dwork C (2011) The promise of differential privacy: A tutorial on algorithmic techniques. In: Proceedings of the 52nd IEEE Annual Symposium on Foundations of Computer Science. pp. 1–12.

4. Fung BCM, Wang K, Chen R, Yu PS (2010) Privacy-preserving data publishing: A survey of recent developments. ACM Computing Surveys 42: 14.

5. US Dept of Health and Human Services (2000) Standards for privacy and individually identifiable health information; final rule. Federal Register 65: 82462–82829.

6. El Emam K, Jabbouri S, Sams S, Drouet Y, Power M (2006) Evaluating common de-identification heuristics for personal health information. Journal of Medical Internet Research 8: e28.

7. El Emam K, Dankar FK, Issa R, et al. (2009) A globally optimal k-anonymity method for the de-identification of health data. Journal of the American Medical Informatics Association 16: 670–682.

8. Loukides G, Gkoulalas-Divanis A, Malin B (2010) Anonymization of electronic medical records for validating genome-wide association studies. Proc Natl Acad Sci USA 107: 7898–78903.

9. Xia W, Heatherly R, Ding X, Li J, Malin B (2013) Efficient discovery of de-identification policy options through a risk-utility frontier. In: Proceedings of the 3rd ACM Cnference on Data and Application Security and Privacy. ACM, pp. 59–70.

10. Sweeney L (2002) k-anonymity: A model for protecting privacy. Int J Uncertainty Fuzz 10: 557–570.

11. Domingo-Ferrer J, Torra V (2005) Ordinal, continuous and heterogeneous k-anonymity through microaggregation. Data Min Knowl Discov 11: 195–212.

12. Sweeney L (2002) Achieving k-anonymity privacy protection using generalization and suppression. Int J Uncertainty Fuzz 10: 571–588.

13. Samarati P, Sweeney L (1998) Protecting privacy when disclosing information: k-anonymity and its enforcement through generalization and suppression. Technical Report Technical Report SRI-CSL-98-04, SRI Computer Science Laboratory.

14. Iyengar VS (2002) Transforming data to satisfy privacy constraints. In: Proc. 8th ACM SIGKDD Int'l Conf. on Knowledge Discovery and Data Mining. pp. 279–288.

15. LeFevre K, DeWitt DJ, Ramakrishnan R (2006) Mondrian multidimensional k-anonymity. In: Data Engineering, 2006. ICDE'06. Proceedings of the 22nd International Conference on. IEEE, pp. 25–25.

16. Elliot M, Dale A (1999) Scenarios of attack: the data intruder's perspective on statistical disclosure risk. Netherlands Official Statistics 14: 6–10.

17. Dankar FK, El Emam K (2010) A method for evaluating marketer re-identification risk. In: Proceedings of the EDBT/ICDT Workshops. ACM, p. 28.

18. Banerjee M, Adl RK, Wu L, Barker K (2011) Quantifying privacy violations. In: Proccedings of the 8th Secure Data Management Workshop, Springer. pp. 1–17.

19. Lebanon G, Scannapieco M, Fouad MR, Bertino E (2006) Beyond k-anonymity: A decision theoretic framework for assessing privacy risk. In: Privacy in Statistical Databases. Springer, pp. 217–232.

20. Benitez K, Malin B (2010) Evaluating re-identification risks with respect to the hipaa privacy rule. Journal of the American Medical Informatics Association 17: 169–177.

21. Tambe M, Jain M, Pita JA, Jiang AX (2012) Game theory for security: Key algorithmic principles, deployed systems, lessons learned. In: Allerton Conference. pp. 1822-1829.

22. An B, Brown M, Vorobeychik Y, Tambe M (2013) Security games with surveillance cost and optimal timing of attack execution. In: Proceedings of the International Conference on Autonomous Agents and Multi-agent Systems. pp. 223–230.

23. Kargupta H, Das K, Liu K (2007) Multi-party, privacy-preserving distributed data mining using a game theoretic framework. In: Procedings of the 11th European Conference on Principles and Practice of Knowledge Discovery in Databases, Springer. pp. 523–531.

24. Rajbhandari L, Snekkenes E (2011) Using game theory to analyze risk to privacy: An initial insight. In: Fischer-Hübner S, Duquenoy P, Hansen M, Leenes R, Zhang G, editors, Privacy and Identity Management for Life, Springer Berlin Heidelberg, volume 352 of *IFIP Advances in Information and Communication Technology*. pp. 41–51. doi:10.1007/978-3-642-20769-3_4. URL `http://dx.doi.org/10.1007/978-3-642-20769-3_4`.

25. Gianini G, Damiani E (2008) A game-theoretical approach to data-privacy protection from context-based inference attacks: A location-privacy protection case study. In: Proccedings of the 5th Secure Data Management Workshop, Springer. pp. 133–150.

26. Brückner M, Scheffer T (2011) Stackelberg games for adversarial prediction problems. In: Proceedings of the 17th ACM SIGKDD International Conference on Knowledge Discovery and Data Mining. pp. 547–555.

27. Bruckner M, Scheffer T (2009) Nash equilibria of static prediction games. In: Proceedings of Advances in Neural Information Processing Systems. pp. 171–179.

28. Blocki J, Christin N, Datta A, Procaccia AD, Sinha A (2013) Audit games. In: Proceedings of the 23rd International Joint Conference on Artificial Intelligence. pp. 41–47.

29. Conitzer V, Sandholm T (2006) Computing the optimal strategy to commit to. In: Proceedings of the 7th ACM conference on Electronic commerce. ACM, pp. 82–90.
